# Supplementary material for: High-resolution AFM structure of DNA G-wires in aqueous solution
Source: Nat Commun. 2018 May 17;9:1959. doi: 10.1038/s41467-018-04016-y (PMC5958085; doi:10.1038/s41467-018-04016-y)
Supplement: Supplementary file 1 — Supplementary Information [file 41467_2018_4016_MOESM1_ESM.pdf]

High-resolution AFM structure of DNA G-wires in aqueous solution

Krishnashish Bose[1], Christopher J. Lech[1], Brahim Heddi[1,2] and Anh Tuân Phan[1,*]

[1]Division of Physics and Applied Physics, School of Physical and Mathematical Sciences, Nanyang Technological University, Singapore

[2]Present address: Laboratoire de Biologie et de Pharmacologie Appliquée, UMR 8113 CNRS, Ecole Normale Supérieure Paris-Saclay, France

*Corresponding authors: phantuan@ntu.edu.sg

SUPPLEMENTARY INFORMATION

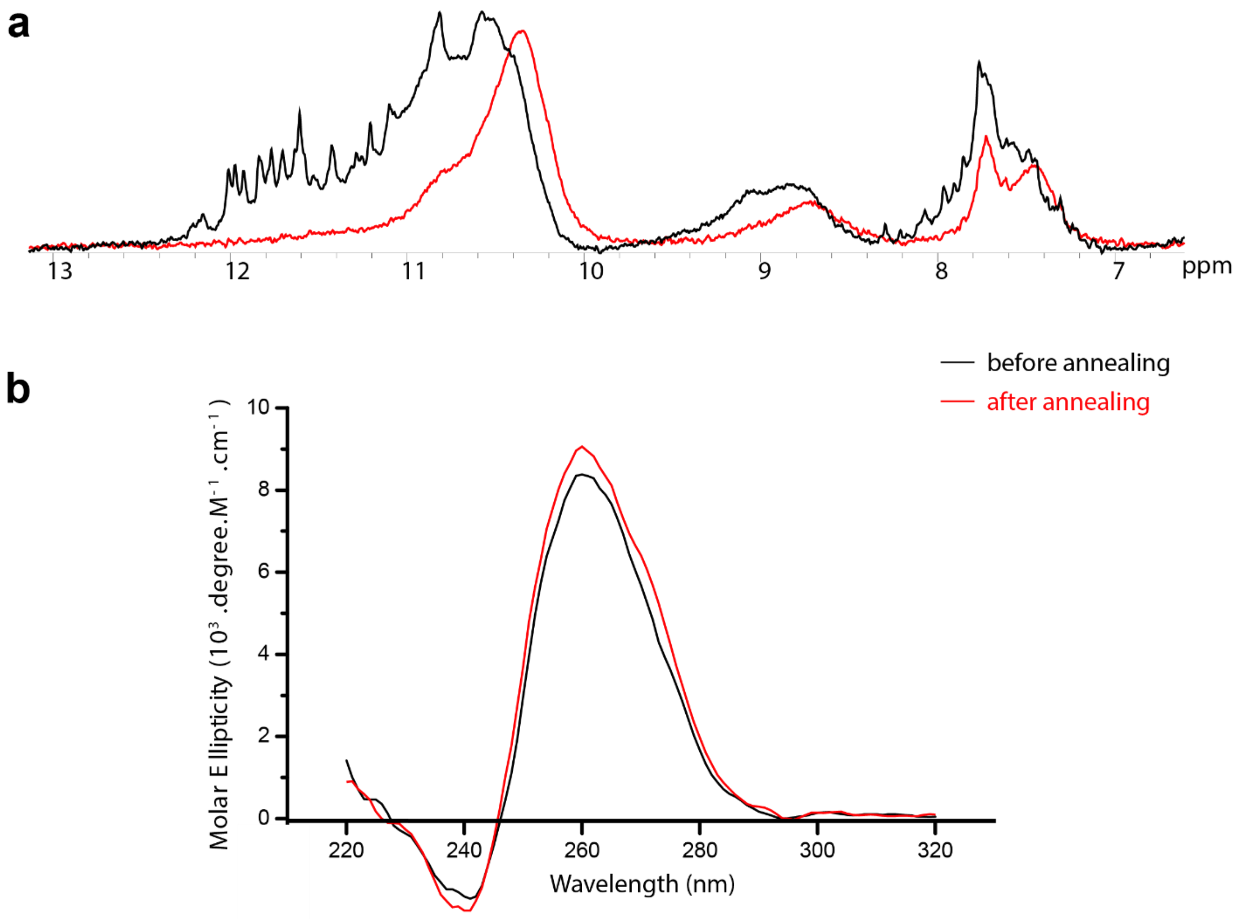

Supplementary Figure 1 | NMR and CD spectroscopy

(a) Proton NMR and **(b)** CD spectra of d[$G_4T_2G_4$] before and after annealing in 30 mM KPi buffer (pH 7) with 70 mM KCl and 10 mM $MgCl_2$.

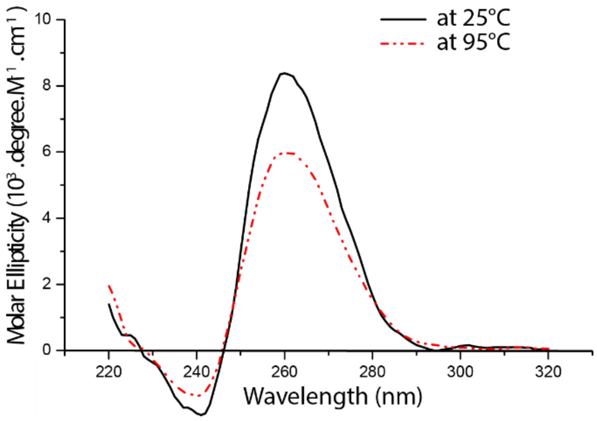

Supplementary Figure 2 | Stability of parallel G-quadruplexes formed by d[$G_4T_2G_4$]

CD spectra obtained for d[$G_4T_2G_4$] at 25°C and at 95°C. The intensity of the peak at 260 nm diminishes by only ~30%.

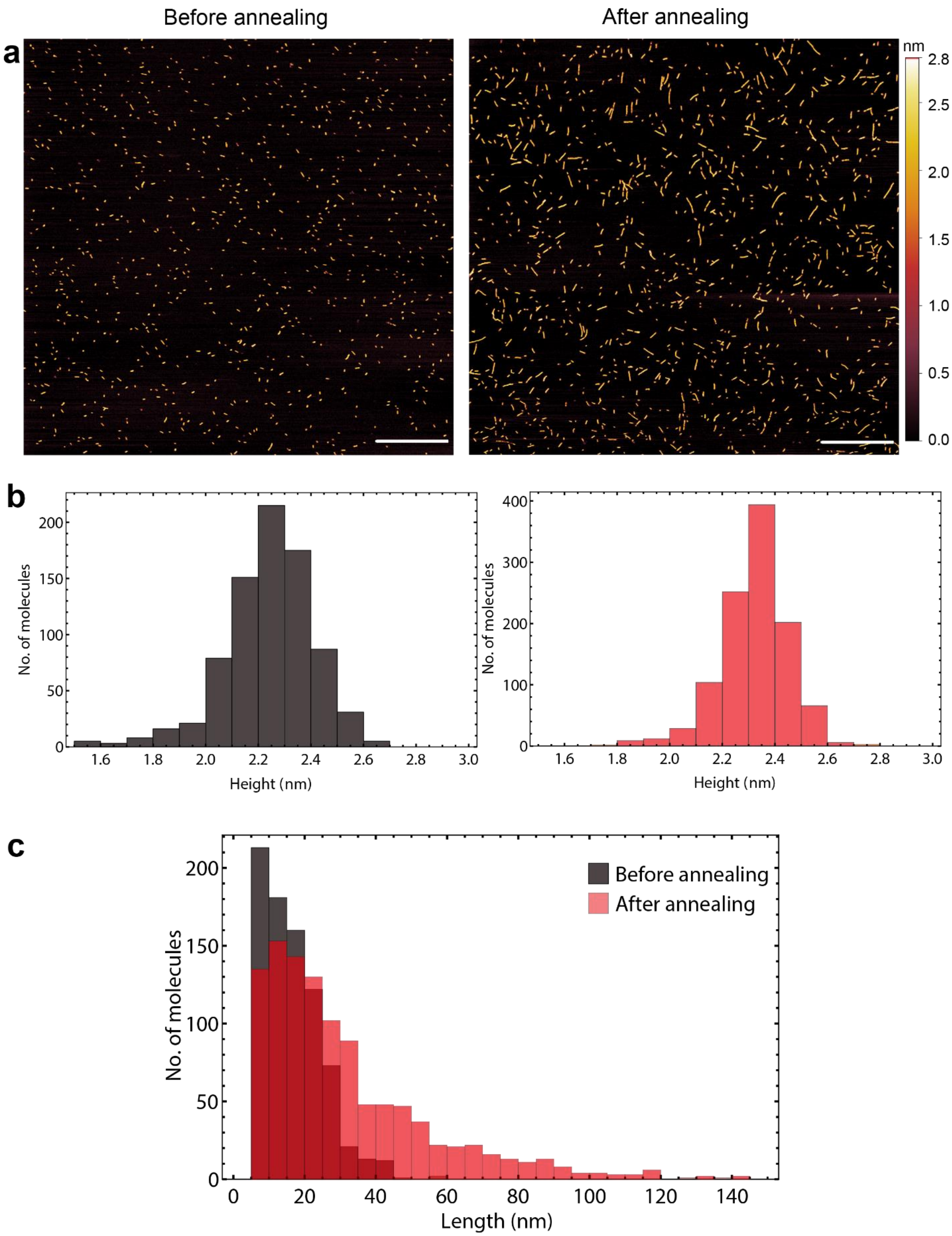

Supplementary Figure 3 | d[$G_4T_2G_4$] before and after annealing

(a) AFM images obtained in air for d[$G_4T_2G_4$] before and after annealing. The stock samples had the same DNA concentration (190 μM) and buffer conditions (90 mM KCl, 10 mM $MgCl_2$ and 20 mM HEPES at pH 7). For AFM imaging both samples were diluted to 19 μM in AFM buffer containing 10 mM $MgCl_2$ and 5 mM HEPES. 0.5-2 μL sample was further diluted with 5 μL of the same AFM buffer just before putting on freshly cleaved Mica. It was rinsed with running water and dried before imaging. Length scale (white bar) is 500 nm. **(b)** Height histogram obtained from the above image show similar height for both samples, but slightly more uniform height after annealing. **(c)** The length histogram was obtained from the spline of all molecules in the image having length greater than 5 nm. Note the overlap of the two length distributions for sample before annealing (black) and after annealing (light red) giving rise to a dark red color. Annealing of the sample clearly led to the formation of longer wires.

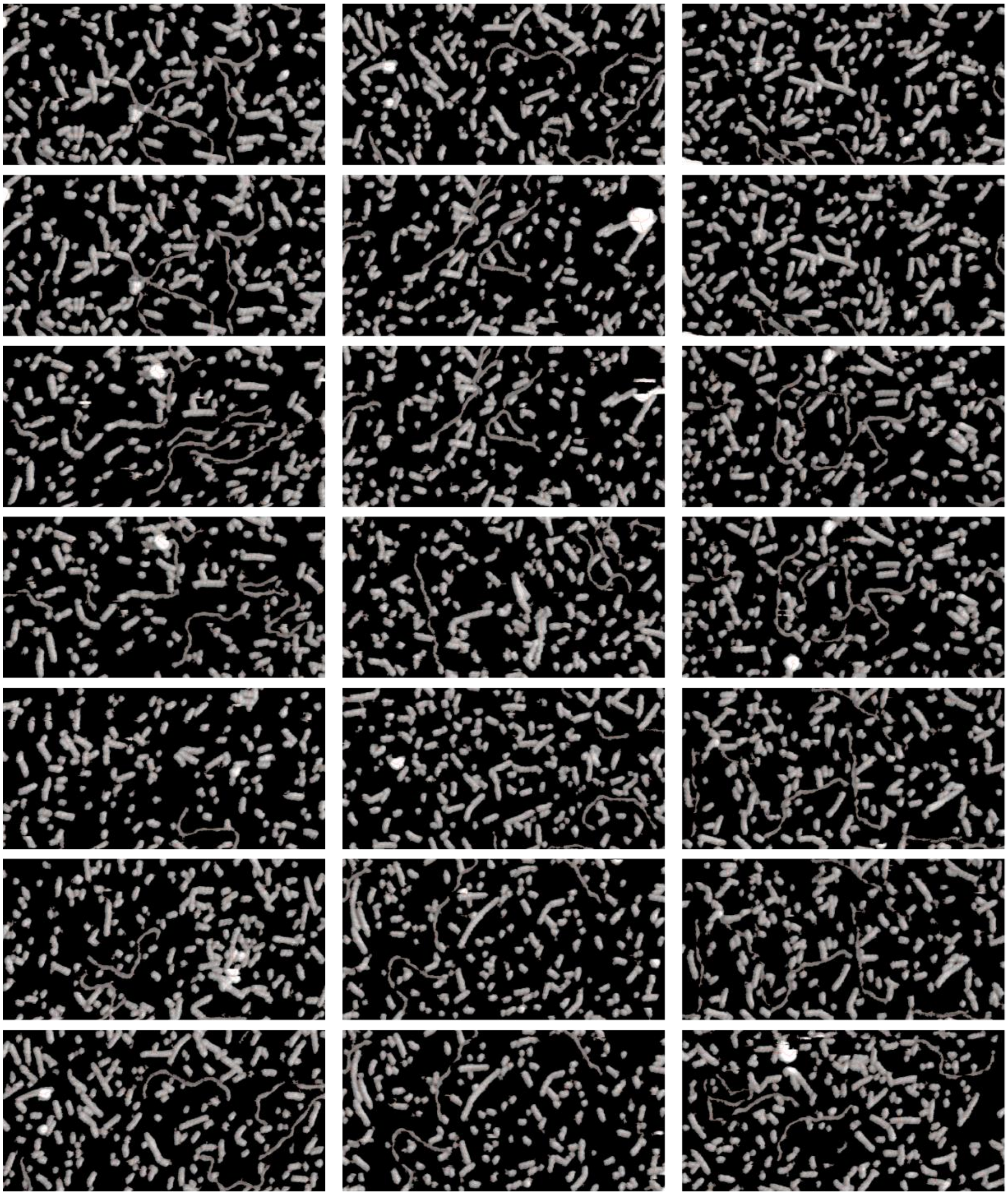

Supplementary Figure 4 | Molecules and their splines from which height was analyzed

High-resolution topography images of G-wires and duplex DNA used for determination of height distribution of the molecules. The central spline of the molecules was detected after removing the background using pixel count threshold of 1000 and Otsu's intensity threshold. The grayscale range of each image is 5 nm with the maximum roughly about 4.5 nm in actual value.

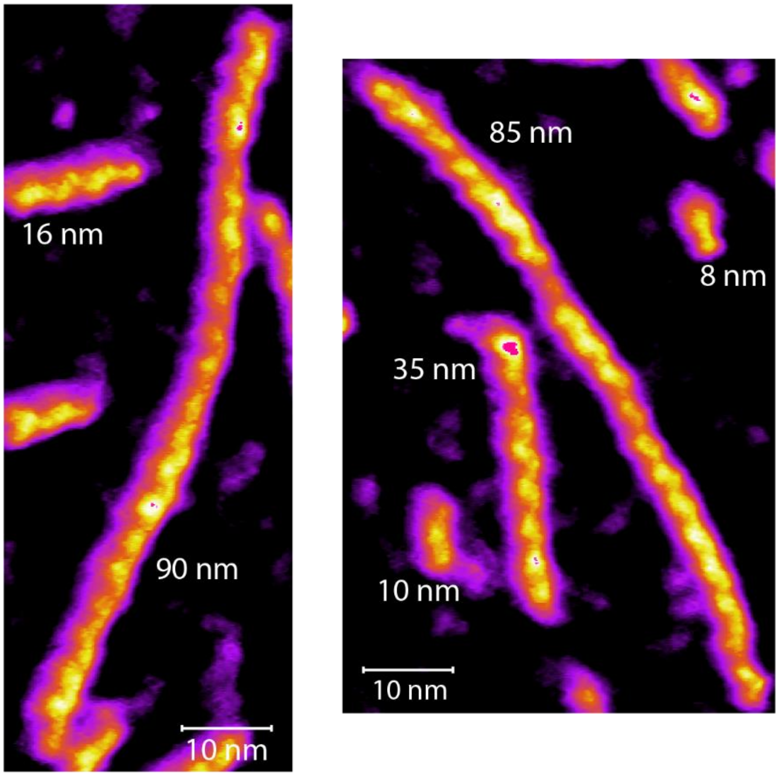

Supplementary Figure 5 | G-wires of various lengths

8 – 90 nm DNA G-wires of d[$G_4T_2G_4$] observed in solution.

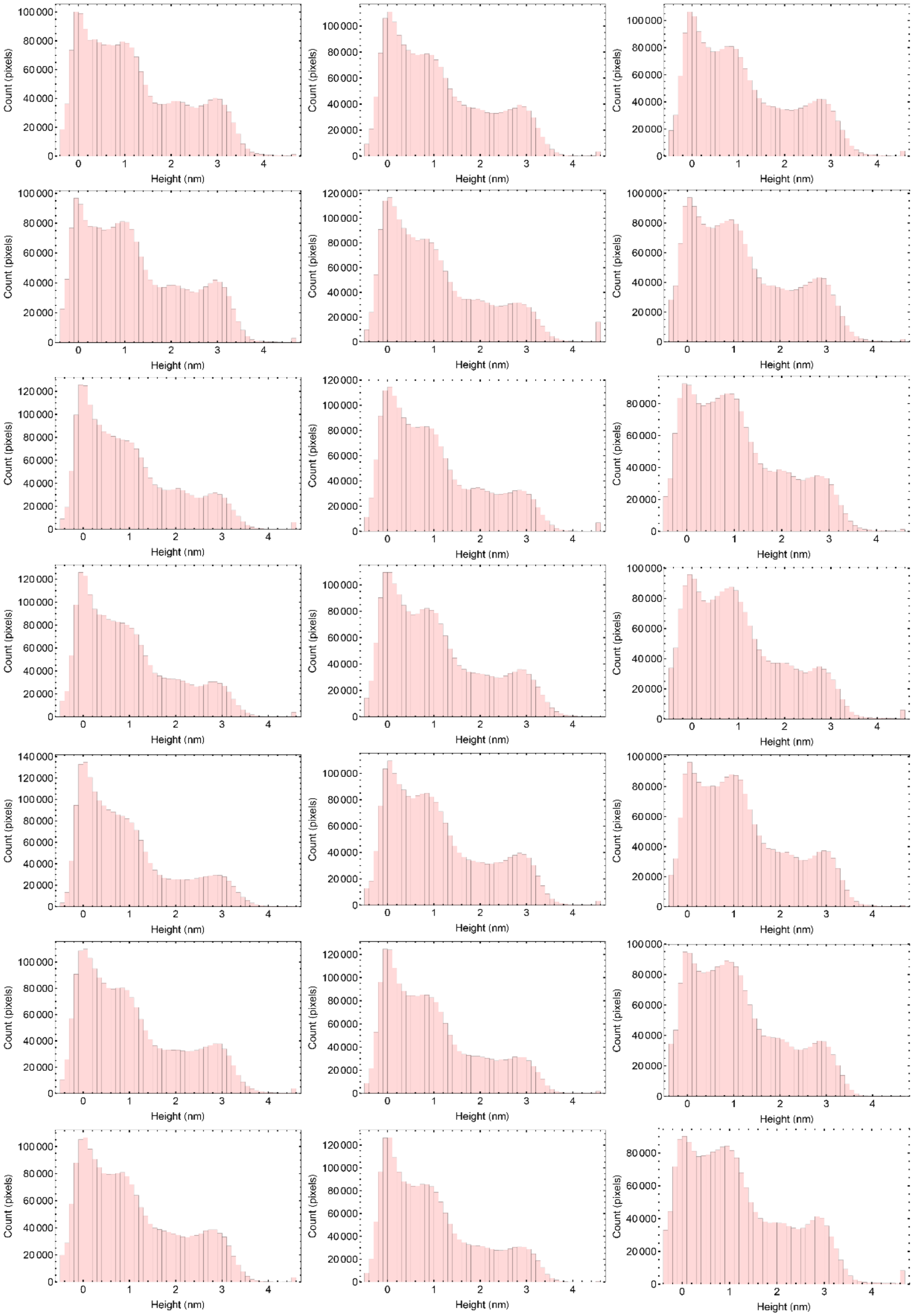

Supplementary Figure 6 | Height histogram of images with duplex DNA and G-wires

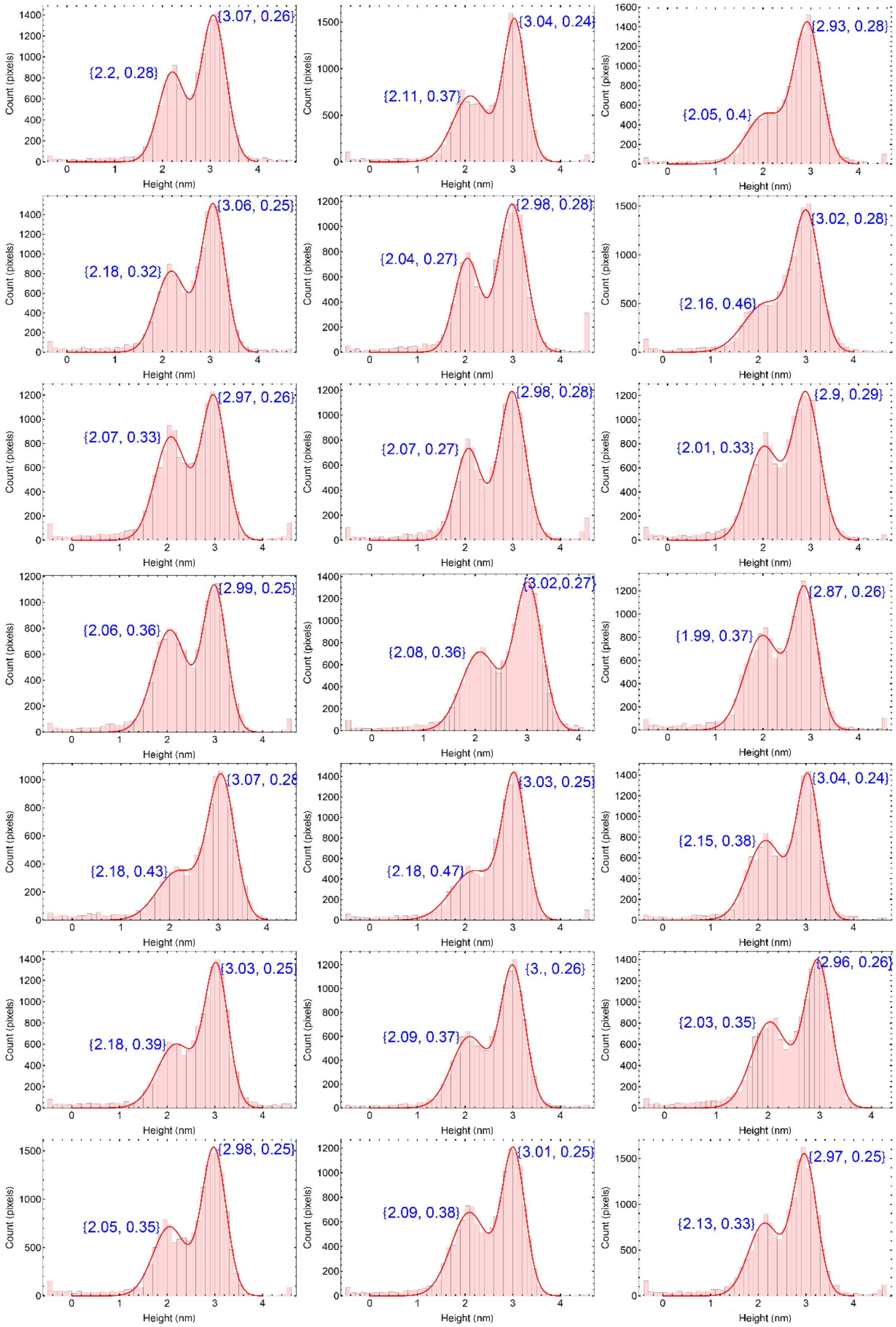

Supplementary Figure 7 | Height histogram from splines of duplex DNA and G-wires

The values shown in blue are the Gaussian fitted mean and standard deviation respectively. In all 21 height histograms obtained from the thinned contours (splines), two distinct peaks were observed, which supposedly corresponded to duplex DNA and G-wires. The values obtained from the histograms by fitting a double Gaussian are shown, from which height differences were calculated as 0.9±0.03 nm: [0.87, 0.88, 0.9, 0.93, 0.89, 0.85, 0.93, 0.93, 0.94, 0.91, 0.94, 0.85, 0.91, 0.92, 0.88, 0.86, 0.89, 0.88, 0.89, 0.93, 0.84] nm.

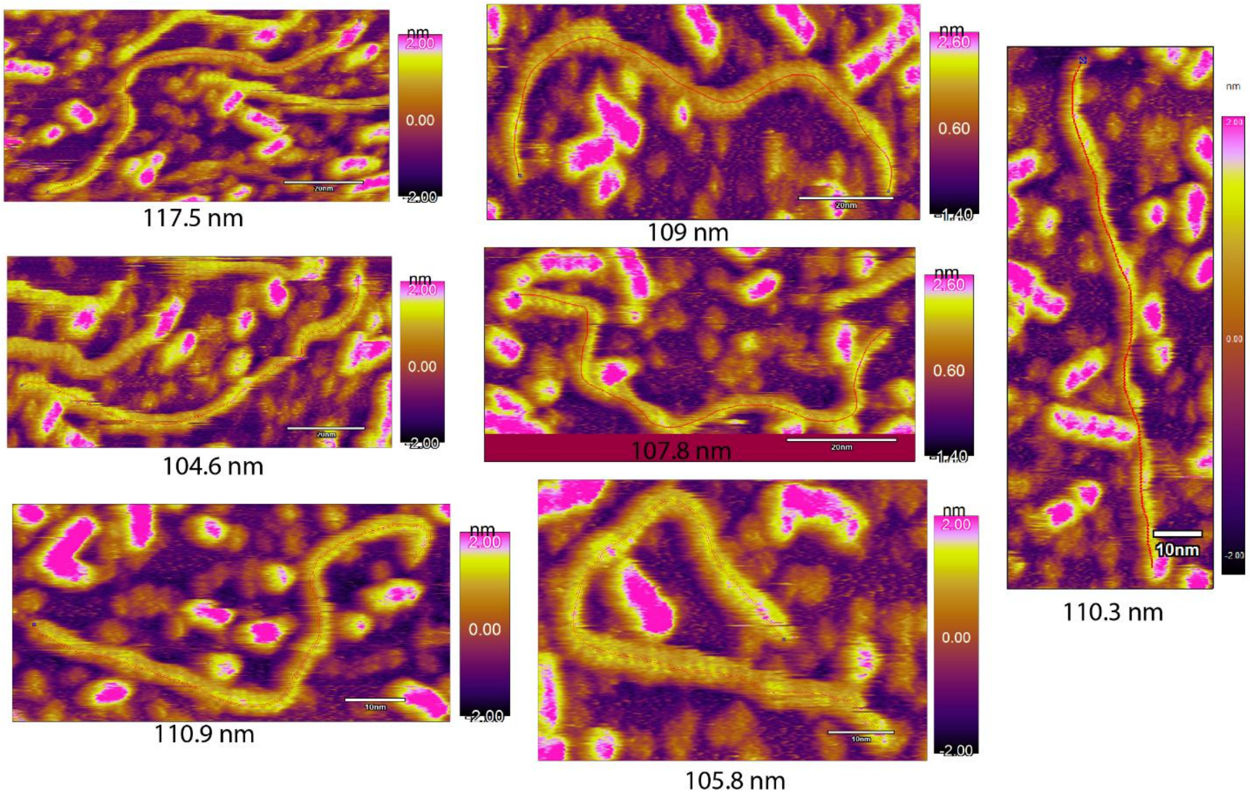

Supplementary Figure 8 | Contour lengths of duplex DNA

Length of duplex DNA from AFM images agree roughly with the expected length of 109 nm for 320 base pairs. Asylum research's licensed AFM software was used to trace the curved splines along the molecule manually from the AFM images obtained together with G-wires.

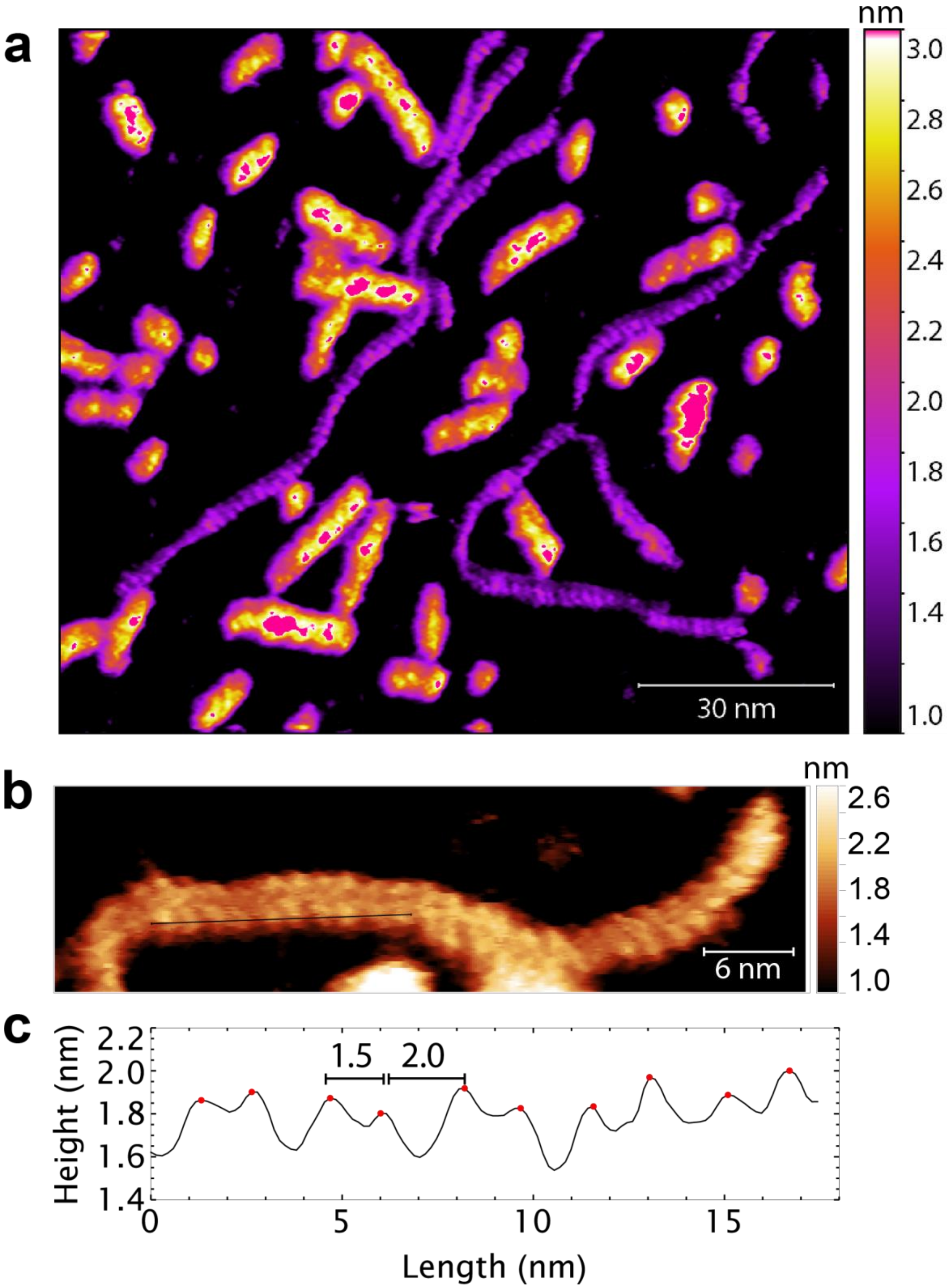

Supplementary Figure 9 | Grooves of duplex DNA

(a) AFM image containing both well-resolved G-wires and duplex DNA. **(b)** A section of duplex DNA zoomed in to show the minor and major grooves. **(c)** Height profile along a selected portion show the width of minor and major grooves as 1.5 nm and 2.0 nm respectively.

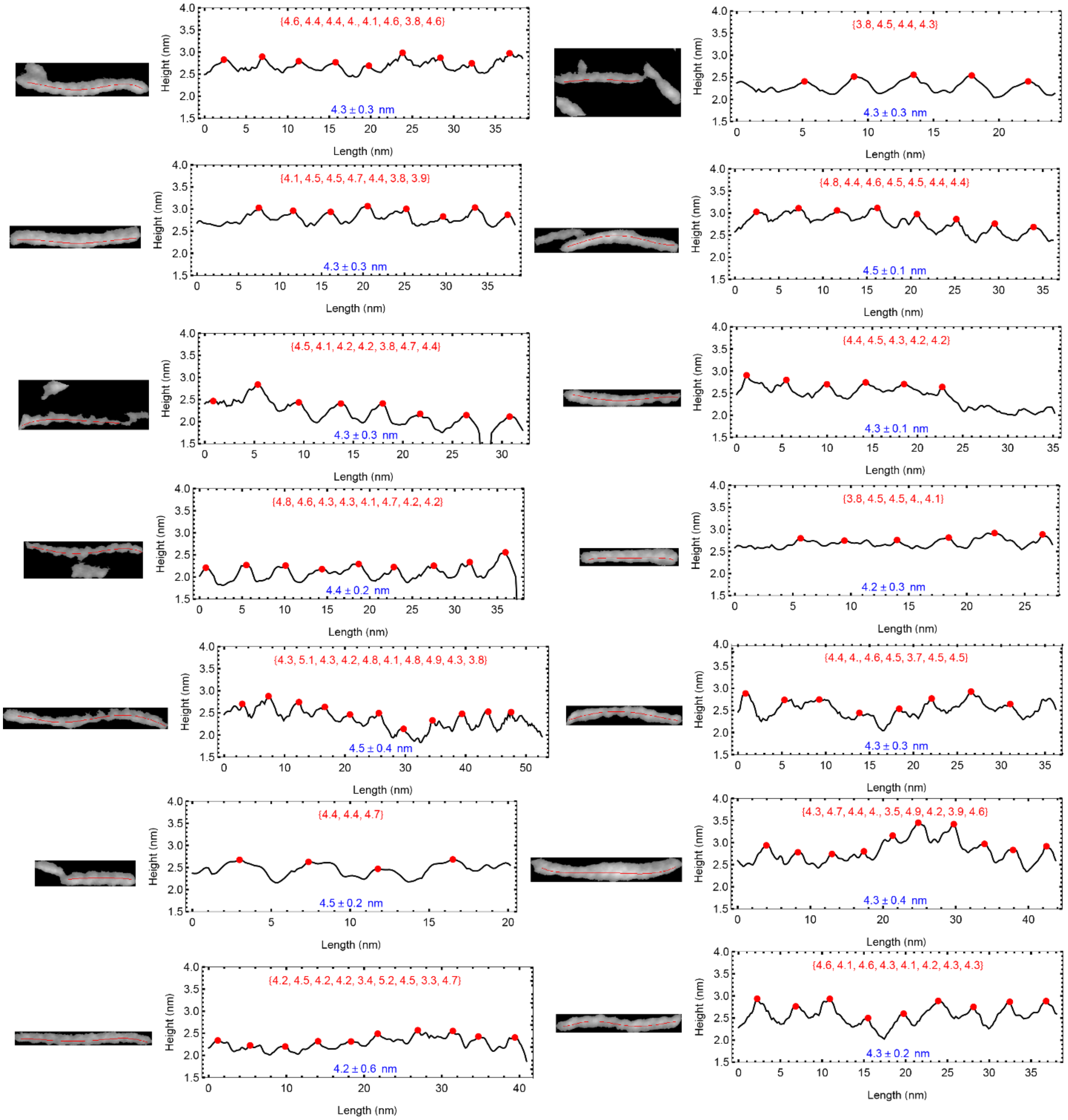

Supplementary Figure 10 | Grooves of *Type I* G-wires

Examples of high-resolution images of *Type I* G-wires and height profile along the highlighted spline with peaks identified. Distances between consecutive peaks are shown on the profile.

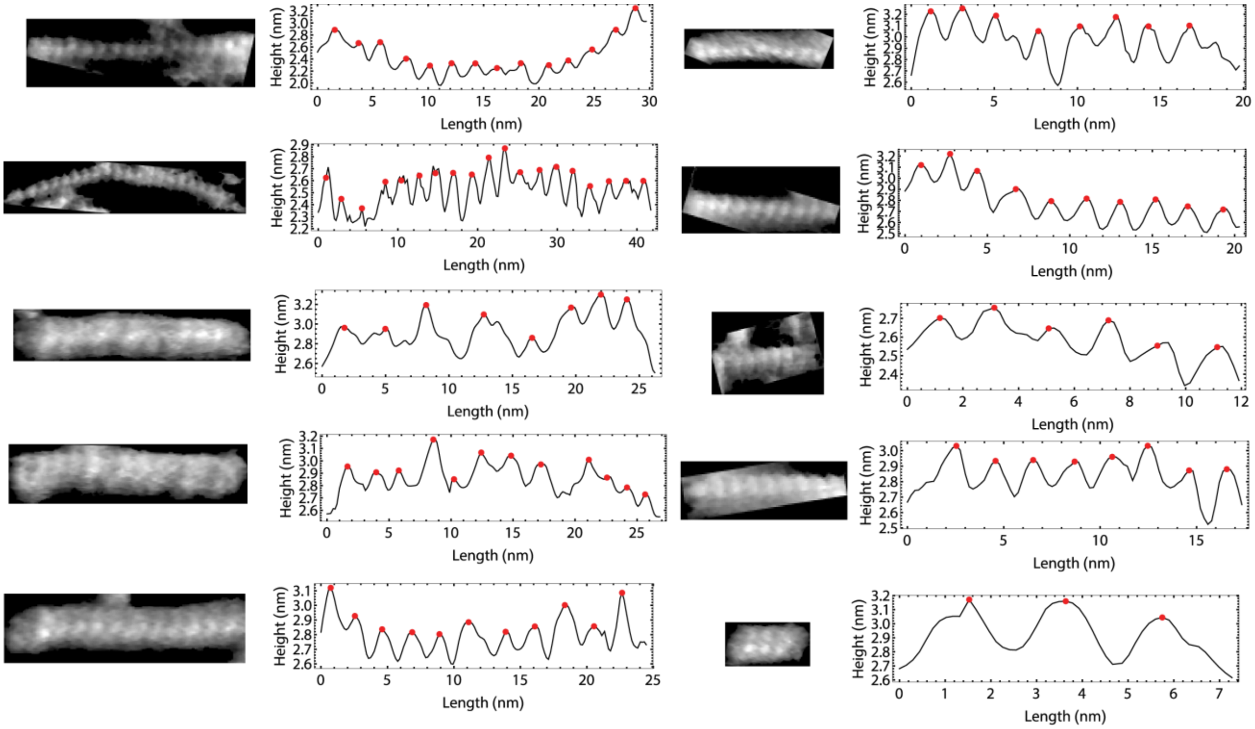

Supplementary Figure 11 | Grooves of *Type II* G-wires

Examples of high-resolution images of *Type II* G-wires and height profile along their entire length.

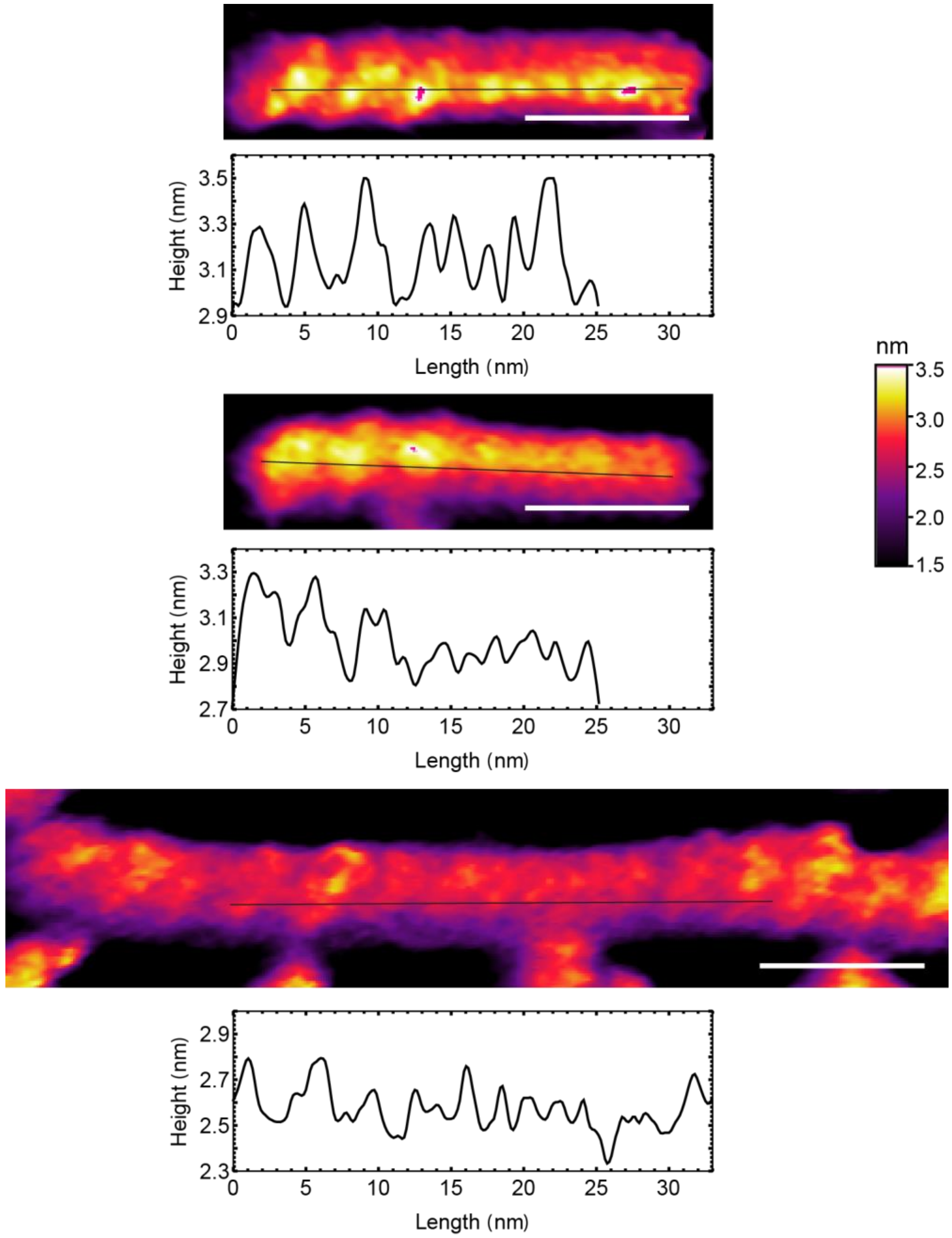

Supplementary Figure 12 | Grooves of *Hybrid Type* G-wires

AFM images of *Hybrid Type* G-wires. The length scale (white bar) is 10 nm. Height profile extracted from the above images.

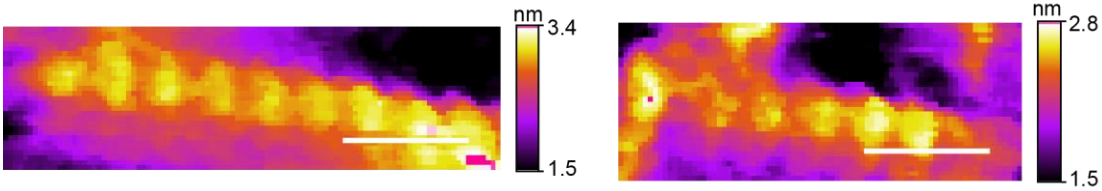

Supplementary Figure 13 | Grooves of *right-handed Type II* G-wires

AFM images of *right-handed Type II* G-wires with the same 2.2 nm periodicity. The length scale (white bar) is 5 nm. It was observed in only 1 image obtained using Biolever mini probe.

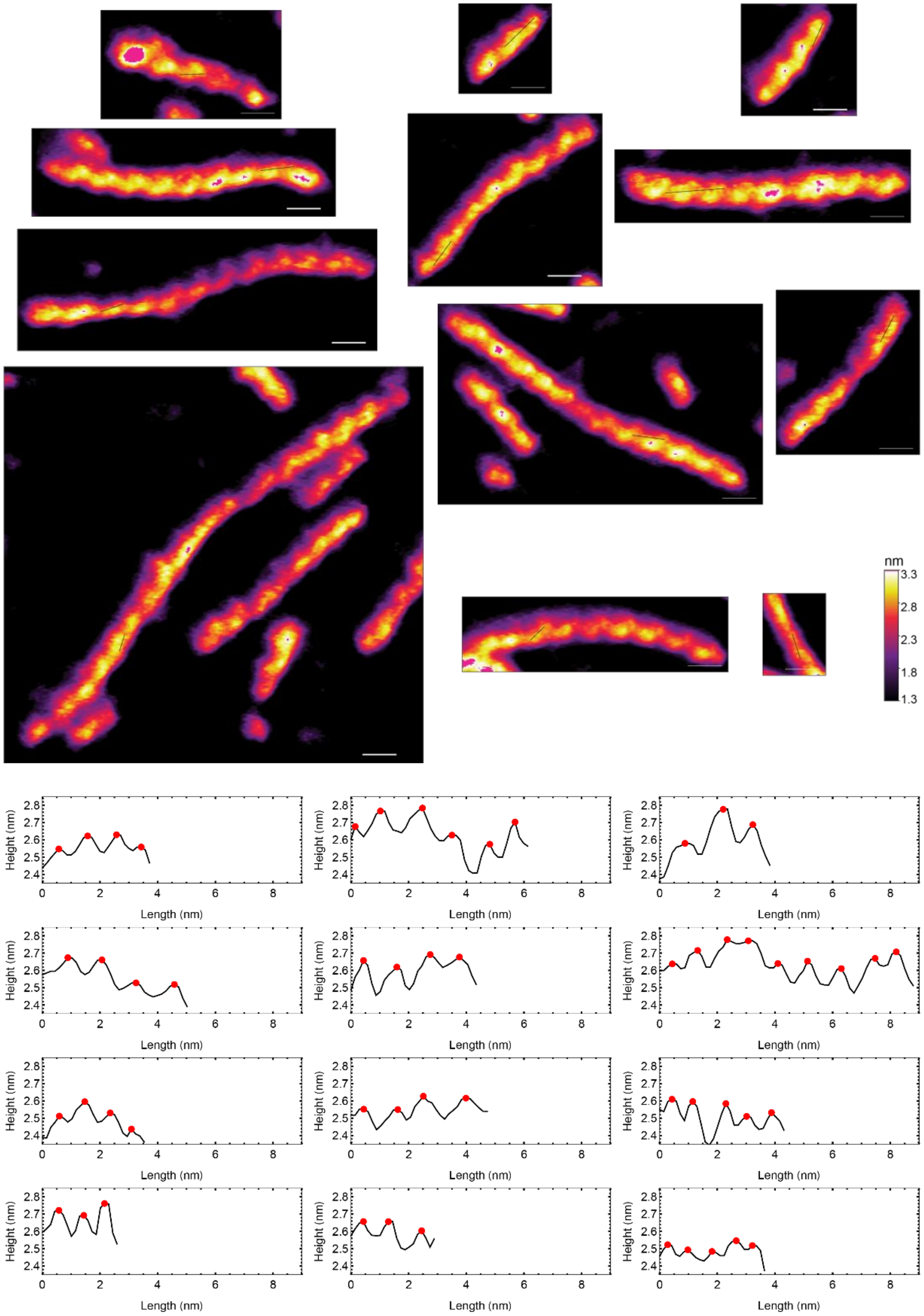

Supplementary Figure 14 | Grooves of *left-handed Type II* G-wires

Type I G-wires which revealed 0.9-nm sub-features in addition to the 4.3-nm features at selected sections (black line). The length scale (white bar) is 5 nm for all images. The height profiles

correspond to the drawn line from which peaks (red dot) were automatically detected to obtain periodicity histogram.

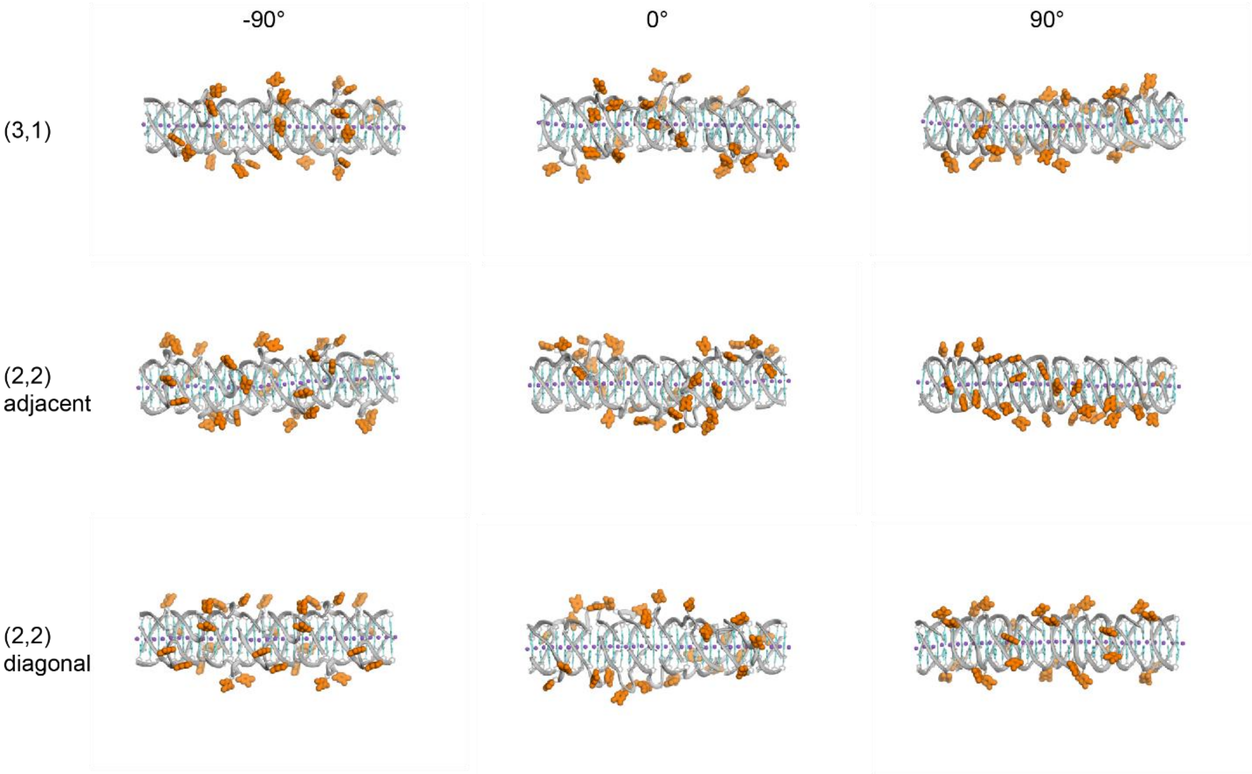

Supplementary Figure 15 | Structure of 9 models

Structure of all 9 models of 11-12 nm length, built using XPLOR and minimized in vacuum with K^+ ions at the core using Amber.

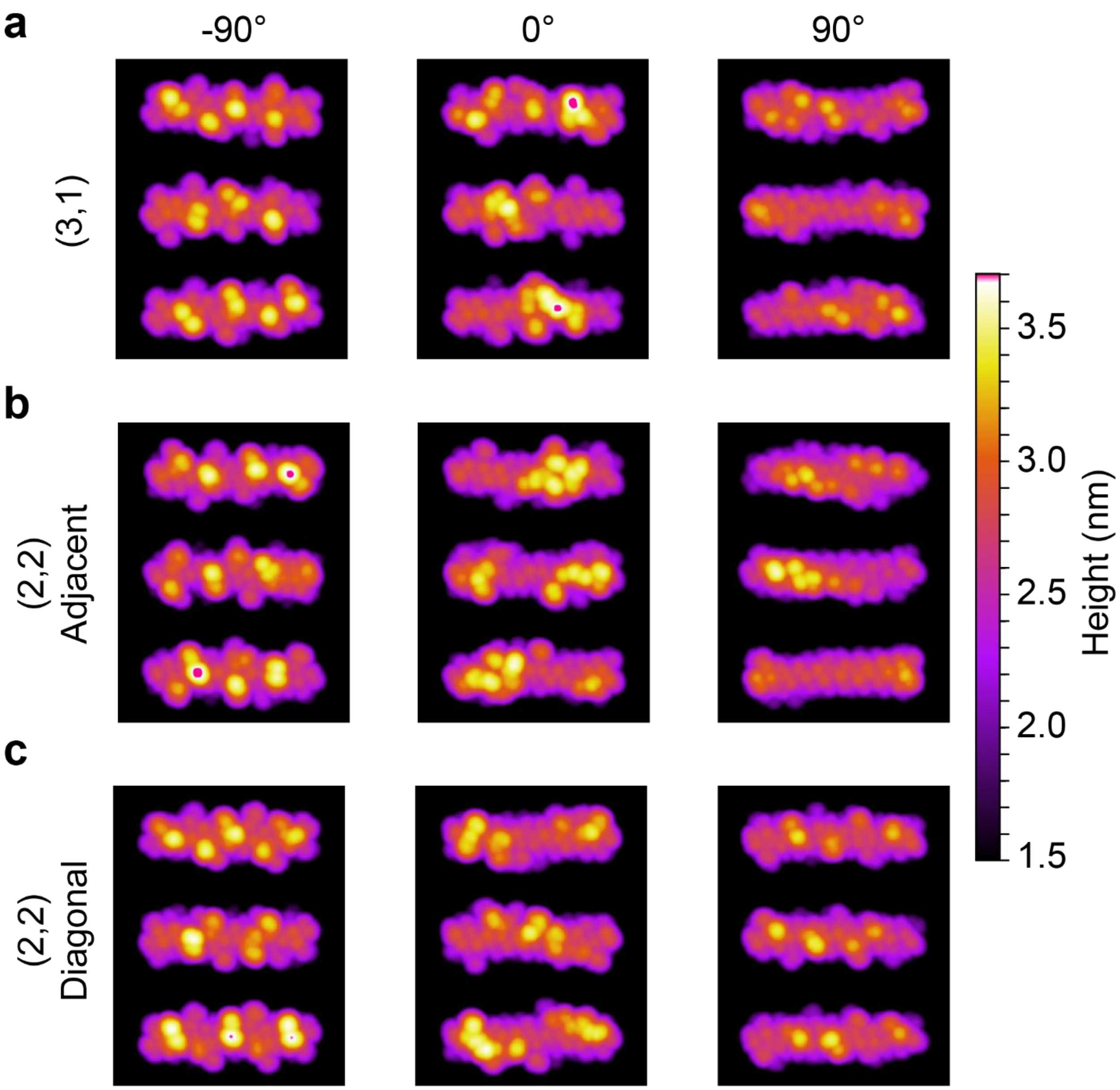

Supplementary Figure 16 | Simulated AFM images

Simulated AFM images of slip-strand G-wire models. Images of the **(a)** *(3,1)* **(b)** *(2,2) Adjacent* and **(c)** *(2,2) Diagonal* arrangements adopting (*left*) -90°, (*center*) 0°, (right) +90° rotamers. Three orientations of the G-wire molecule are shown for each model. Only the *(2,2) Diagonal* arrangement with a -90° rotamer displays clear *Type I* features.

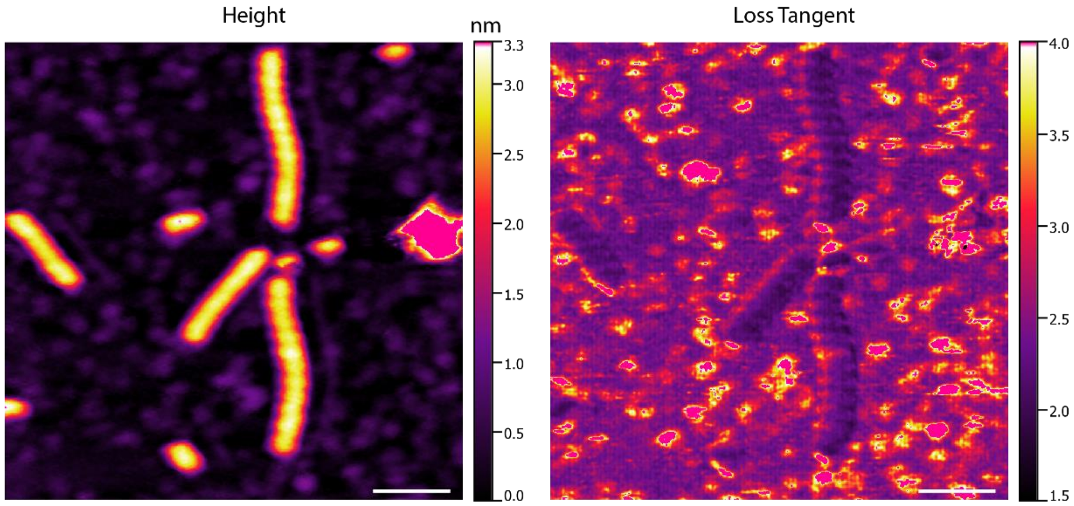

Supplementary Figure 17 | Height vs. Loss Tangent for G-wires

AFM images of G-wires showing periodic pattern in both height and loss tangent. The length scale (white bar) is 10 nm.

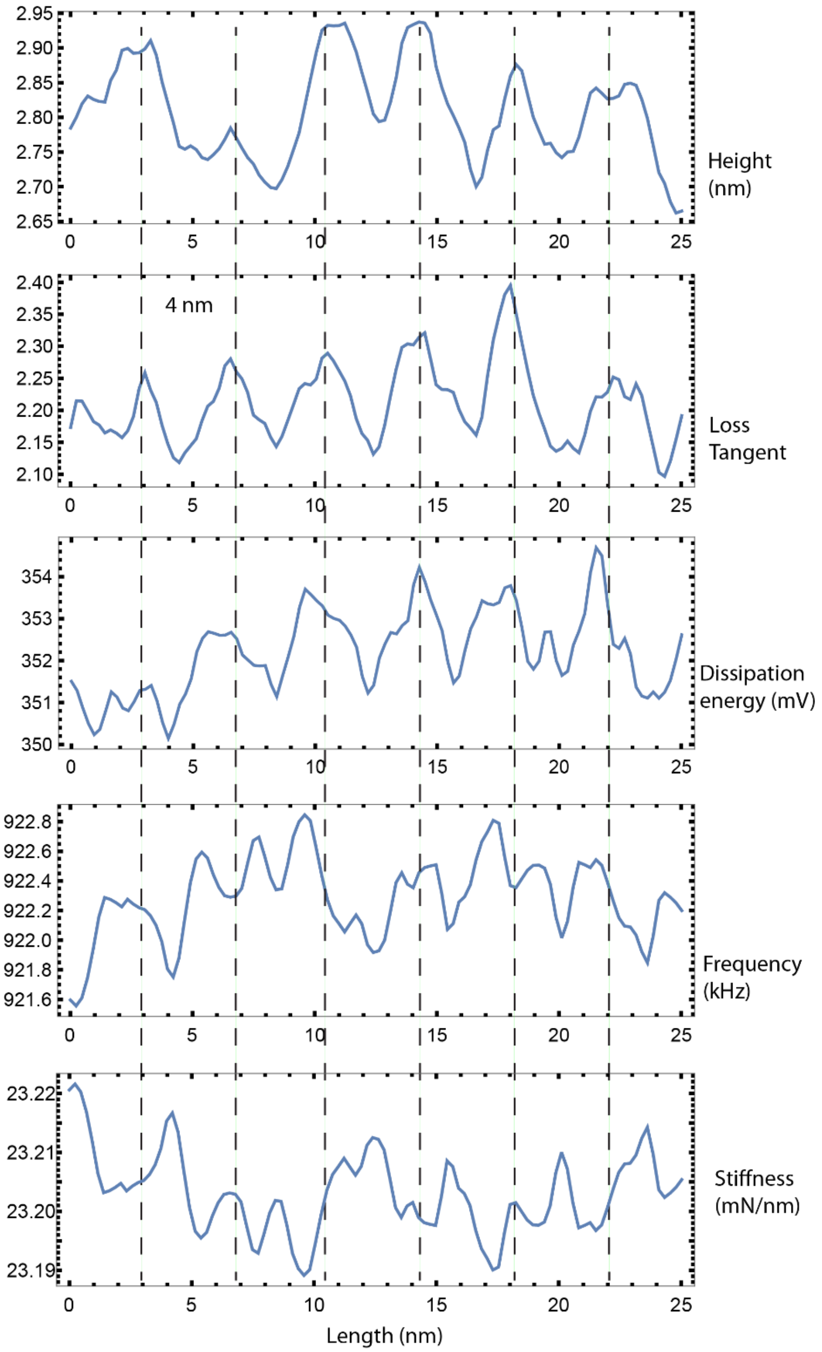

Supplementary Figure 18 | 4 nm periodicity in topography and mechanical properties

A small section of a G-wire was chosen from the above image and the profile was obtained from all channels to correlate different mechanical properties and topography. The dotted lines are all separated by 4 nm and correlate directly with peaks in height, loss tangent and dissipation energy. For the frequency and stiffness channel, the pattern is a bit more complicated. The individual peaks in height was found to split into two or three peaks in frequency/stiffness.

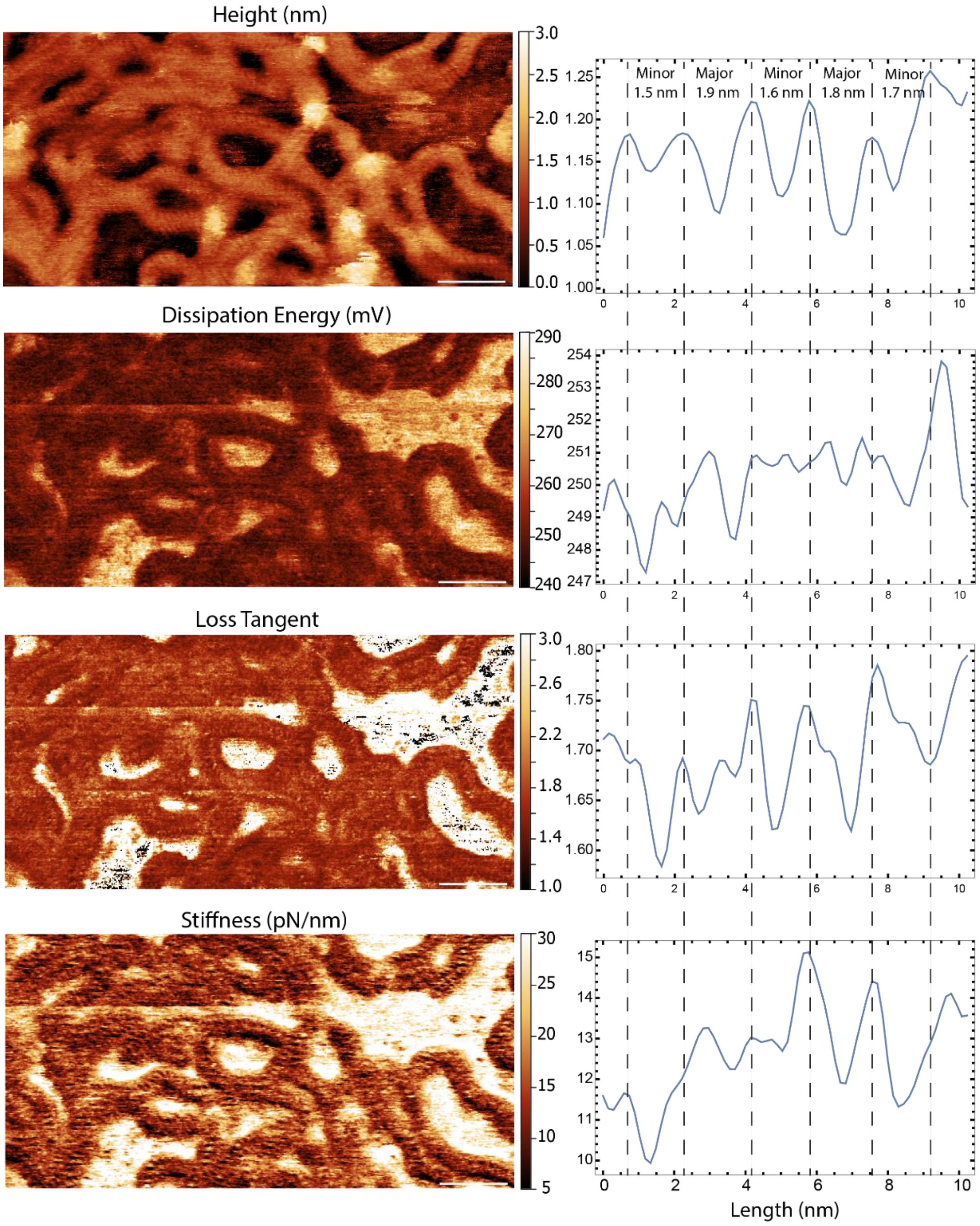

Supplementary Figure 19 | Mechanical mapping of minor and major grooves of duplex DNA

Resolving the minor and major grooves of supercoiled plasmid DNA in height, dissipation energy, loss tangent and stiffness channels simultaneously. The image was obtained in AM-FM mode using FastScan-D. The length scale (white bar) is 30 nm. A small section was chosen in the image and the profile was obtained from all channels to correlate different mechanical properties and topography.

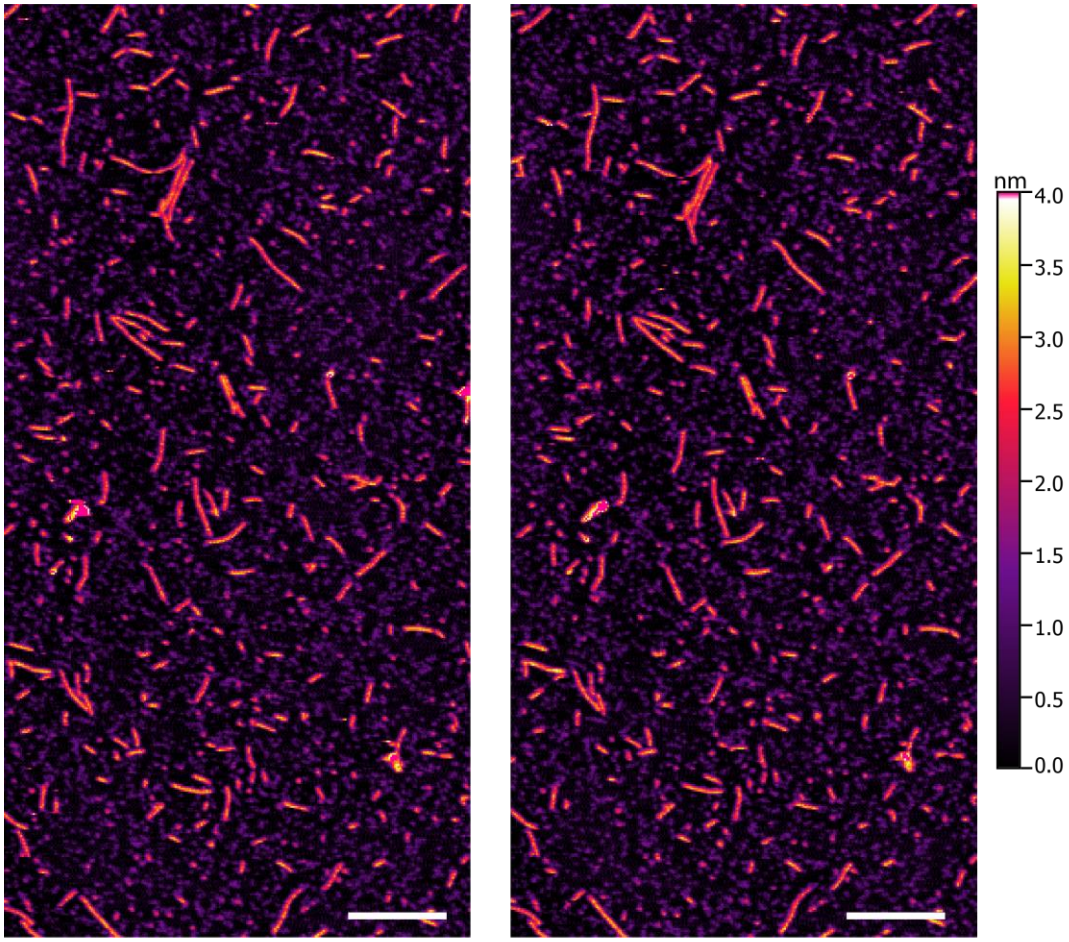

Supplementary Figure 20 | Reproducible imaging of G-wires

Images obtained from successive scanning of the same area containing wires of d[G_4TTG_4] in solution on Mica. The purple background is likely due to excessive $NiCl_2$ salt precipitated on the Mica surface. The image was obtained in AM-FM mode using FastScan-D. The length scale (white bar) is 100 nm.

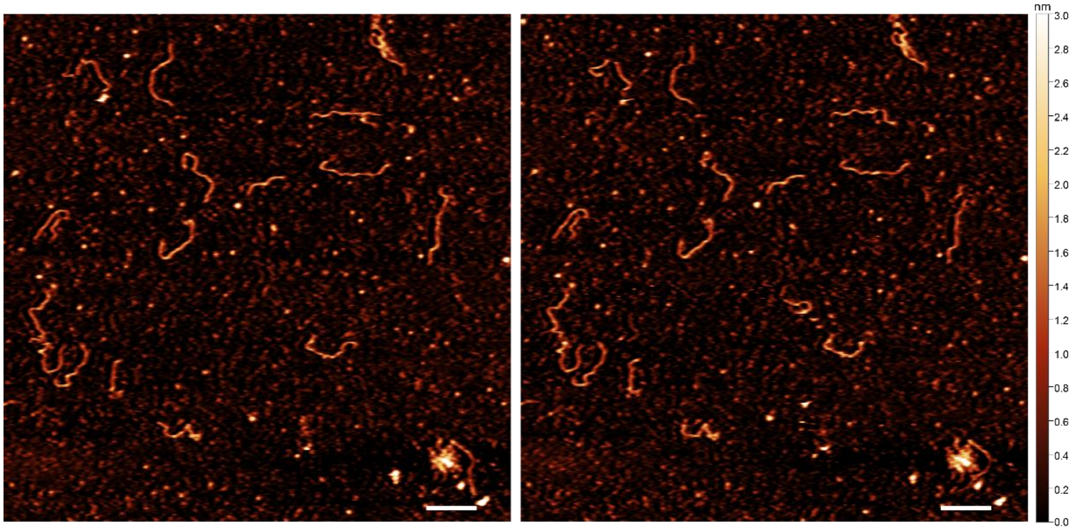

Supplementary Figure 21 | Reproducible imaging of duplex DNA with just 0.2 mM $NiCl_2$

Images obtained from successive scanning of the same area containing duplex DNA (0.2 ng/µL). With just 0.2 mM $NiCl_2$, it was possible to immobilize duplex DNA for high-speed imaging using FastScan-D probe in AM mode. The length scale (white bar) is 100 nm.

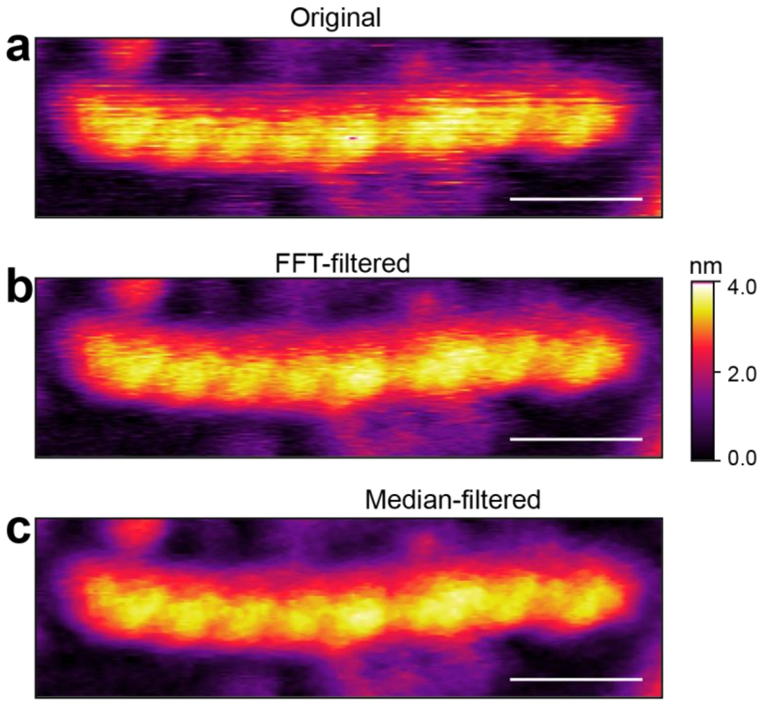

Supplementary Figure 22 | Proper filtering of high-resolution AFM images

A representative *Type I* G-wire at different stages of filtering shows that the relevant periodic information was preserved with FFT filtering, while only the high frequency noise was removed. A median filter of 3 pixels ensured that the image was smoothened properly so that spurious peaks are not encountered during periodicity analysis. The length scale (white bar) is 5 nm.

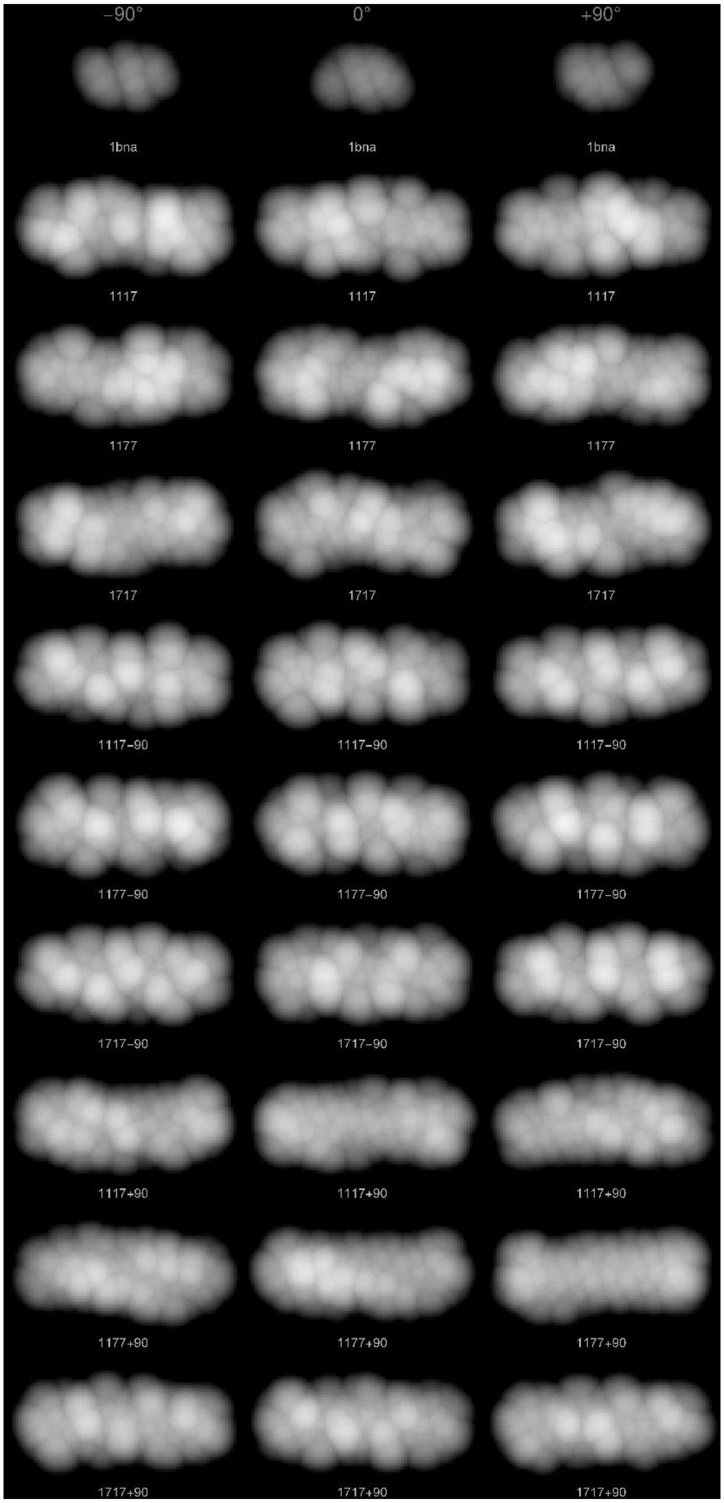

Supplementary Figure 23 | Simulated AFM images with a sharper virtual tip

Simulated AFM images of slipped-strand G-wire models. Images of the *(3,1)* (or 1117), *(2,2) Adjacent* (or 1177) and *(2,2) Diagonal* (or 1717) configurations adopting (*left*) -90°, (*center*) 0°, (right) +90° rotamers. Three orientations of the G-wire molecule are shown for each model.
